# The Low Energy-Coupling Respiration in *Zymomonas mobilis* Accelerates Flux in the Entner-Doudoroff Pathway

**DOI:** 10.1371/journal.pone.0153866

**Published:** 2016-04-21

**Authors:** Reinis Rutkis, Inese Strazdina, Elina Balodite, Zane Lasa, Nina Galinina, Uldis Kalnenieks

**Affiliations:** Institute of Microbiology and Biotechnology, University of Latvia, Jelgavas Street 1, Riga, LV-1004, Latvia; Instituto Nacional de Cardiologia, MEXICO

## Abstract

Performing oxidative phosphorylation is the primary role of respiratory chain both in bacteria and eukaryotes. Yet, the branched respiratory chains of prokaryotes contain alternative, low energy-coupling electron pathways, which serve for functions other than oxidative ATP generation (like those of respiratory protection, adaptation to low-oxygen media, redox balancing, etc.), some of which are still poorly understood. We here demonstrate that withdrawal of reducing equivalents by the energetically uncoupled respiratory chain of the bacterium *Zymomonas mobilis* accelerates its fermentative catabolism, increasing the glucose consumption rate. This is in contrast to what has been observed in other respiring bacteria and yeast. This effect takes place after air is introduced to glucose-consuming anaerobic cell suspension, and can be simulated using a kinetic model of the Entner-Doudoroff pathway in combination with a simple net reaction of NADH oxidation that does not involve oxidative phosphorylation. Although aeration hampers batch growth of respiring *Z*. *mobilis* culture due to accumulation of toxic byproducts, nevertheless under non-growing conditions respiration is shown to confer an adaptive advantage for the wild type over the non-respiring Ndh knock-out mutant. If cells get occasional access to limited amount of glucose for short periods of time, the elevated glucose uptake rate selectively improves survival of the respiring *Z*. *mobilis* phenotype.

## Introduction

**Fig 1:**
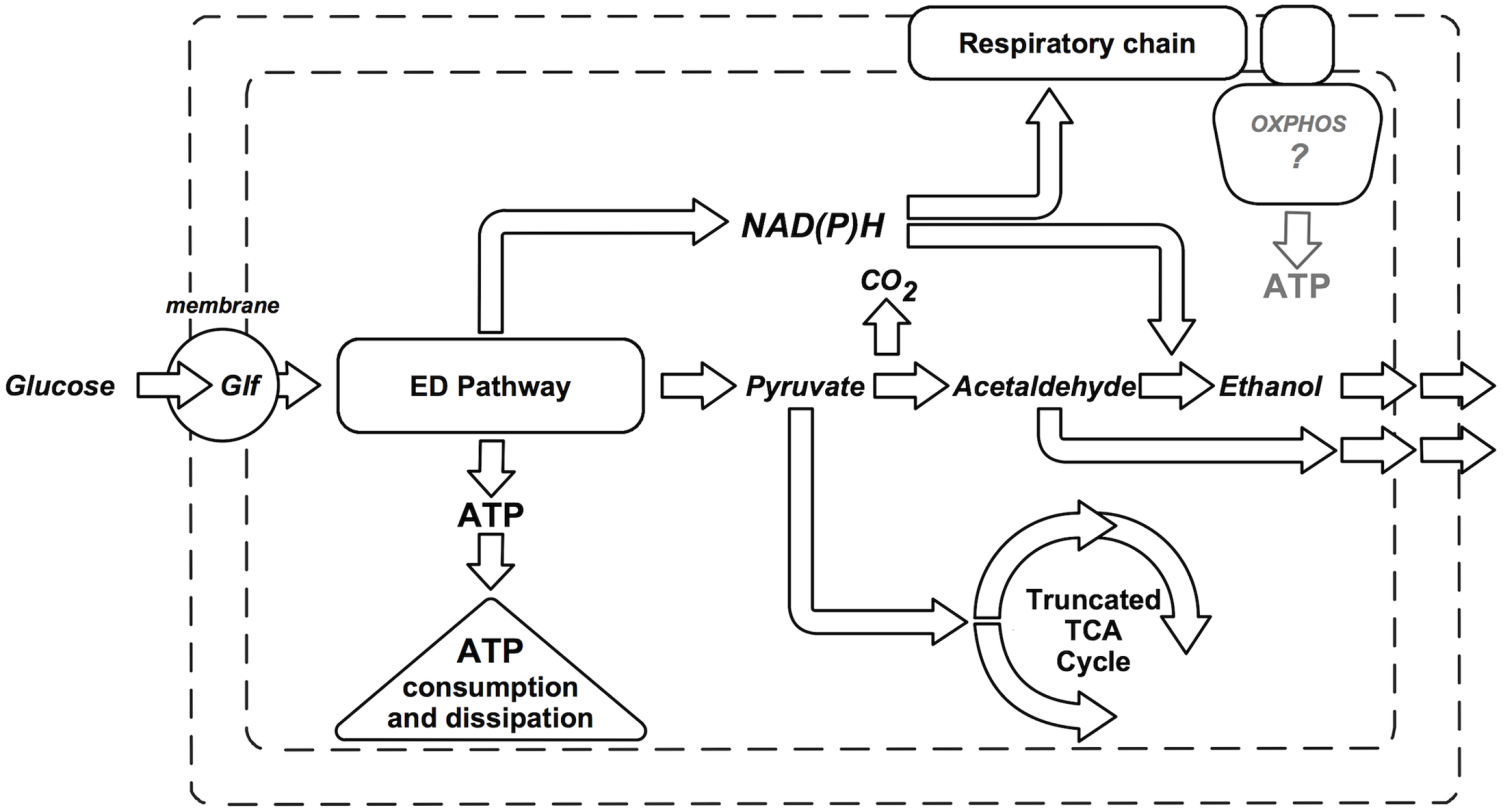
An overview of glucose catabolism in *Z*. *mobilis*.

Bacterium *Zymomonas mobilis* possesses the most rapid ethanologenic pathway among microorganisms [1], which is composed of the Entner–Doudoroff (E-D) glycolytic pathway in combination with pyruvate decarboxylase and two alcohol dehydrogenase isoenzymes. *Z*. *mobilis* is a facultatively anaerobic, obligately fermentative bacterium, able to grow exclusively on glucose, fructose or sucrose as the carbon source. Up to 98% of substrate carbon is incorporated in the catabolic end product ethanol. The outstanding ethanol-synthesizing ability of this bacterium has promoted metabolic engineering work on bioethanol production [2–4]. At the same time, *Z*. *mobilis* membranes contain an active respiratory chain, with the type II NADH dehydrogenase (Ndh), coenzyme Q_10_, and the cytochrome *bd* terminal oxidase as the established major electron carriers, together with some minor or still unidentified constituents [5–11]. Genome sequences of several *Z*. *mobilis* strains are now available [12–15], and *in silico* reconstructions of its metabolic network, as well as genome-scale transcriptomic and proteomic studies have been published during the last decade [16–18]. The genome of *Z*. *mobilis* is relatively small, and the network of central metabolism appears to be simpler than in most model microorganisms, including *Escherichia coli* and *Saccharomyces cerevisiae*. The Krebs cycle in this bacterium is incomplete, and does not operate as a catabolic pathway [19]. Hence, all metabolically available NAD(P)H comes almost exclusively from the E-D pathway. Ethanol synthesis and respiration are the two major sinks of NADH in *Z*. *mobilis* catabolism, competing for the reducing equivalents (Fig 1).

The reasons for evolution of the active respiratory chain in *Z*. *mobilis* remain largely a mystery. For chemotrophic microorganisms, which bear an electron transport chain, the shift from fermentative to respiratory catabolism raises the biomass yield of culture due to the onset of oxidative phosphorylation [20]. Membrane preparations and non-growing cells of *Z*. *mobilis* also show oxidative phosphorylation activity with NAD(P)H or ethanol, respectively, as the electron donors [21]. However, oxidative phosphorylation does not operate in growing *Z*. *mobilis* [22]. Transition from anaerobic to aerobic growth conditions does not improve *Z*. *mobilis* biomass yield [23–25]. The mechanistic reason for the low energy-coupling efficiency in growing *Z*. *mobilis* is not well understood. Generally, respiration in this bacterium lacks a clearly defined physiological function. Presence of the active respiratory chain is not required to survive aerobic conditions. Inhibition of respiration with cyanide [26], or inactivation of the respiratory NADH dehydrogenase (*ndh*) [8, 27, 28] prevents accumulation of the inhibitory metabolite acetaldehyde, and thus even stimulates *Z*. *mobilis* aerobic growth (although does not affect its biomass yield). The ability of *Z*. *mobilis* to fix atmospheric nitrogen was demostrated recently [29], but it is not known whether respiration ensures respiratory protection of the nitrogenase complex, as in *Azotobacter* species [30]. In some aspects, the non-respiring strains of *Z*. *mobilis* appear to be more competitive than the wild type. Under aerobic or microaerobic conditions the non-respiring *ndh* knock-out strain outgrows the wild type, while under anaerobic conditions their growth rates are comparable [8, 27, 28]. Also, the *ndh* mutants show better growth and ethanol production at elevated temperature [28], although appear to be less tolerant to salt stress, than the wild type [31].

Here we examined the interaction between respiration and glucose catabolism, aiming to determine if and how respiration affects the metabolic flux via the E-D pathway. This has been a largely neglected aspect of respiratory function in *Z*. *mobilis* thus far. To approach the problem, we started with model simulations, using the recently developed kinetic model of the *Z*. *mobilis* E-D pathway [32]. The model was complemented with reactions for respiratory catabolism, and the simulation results were compared with experimental data on glucose consumption under anaerobic and aerobic conditions. It appeared that respiration substantially accelerated glucose uptake in non-growing cells, showing a novel role for the bacterial respiratory chain with low energy-coupling. Furthermore, a selective advantage was demonstrated for respiring wild-type *Z*. *mobilis* over non-respiring, *ndh*-deficient cells for survival under conditions of periodic glucose starvation.

## Materials and Methods

### Bacterial strains, cloning and PCR

*Z*. *mobilis* Zm6 (ATCC 29191) and the *Z*. *mobilis* type II respiratory NADH dehydrogenase (*ndh*) gene knock-out mutant Zm6-*ndh* [8] were used in the study. The *ndh* of mutant carried an insert of 1.3 kb with an 0.65 kb fragment encoding the chloramphenicol acetyltransferase gene. For complementation of Zm6-*ndh*, a 1.55 kb chromosomal DNA fragment, containing the ORF of *ndh* together with its promoter region, was amplified by PCR, using the primer pair NADHdeh-1 (AGACAGAGACCGGATCCGGGCAAAGATAG) and NADHdeh-2 (TCGAACGGAAGCTTCCCACCCAAAACAAG). The engineered restriction sites for *Bam*HI and *Hind*III, respectively, are underlined. The amplified fragment was double-digested with *Bam*HI and *Hind*III, and directionally cloned between the *Bam*HI and *Hind*III restriction sites of the MCS of shuttle vector pBBR1MCS-2, yielding plasmid pBBRndh. Plasmid pBBRndh was used for transformation of Zm6-*ndh* by electroporation, to yield the complemented strain Zm6-*ndh*+, as described in [11]. Transformants were selected on agar plates containing chloramphenicol (120 μg ml^-1^) and kanamycin (310 μg ml^-1^). Total DNA of the transformed strains was isolated, and the presence of an intact *ndh* ORF was verified by PCR with primers Z.m.ndh1 (AGAGAATAGAGGGGATCCATGTCGAAGAAT) and Z.m.ndh2 (ATCAGTATAATTAAGCTTTAGGGCGTAACA), followed by sequencing of the amplified product [8, 11]. Colony PCR was used to discriminate between the colonies of Zm6 and Zm6-*ndh*, when mixed cell suspensions of both strains were plated. For that purpose, instead of purified DNA small amount of cells from a colony was added into the PCR assay mix, by using a fine pipette tip. With the primer pair Z.m.ndh1 and Z.m.ndh2, colonies of Zm6 produced a 1.3 kb band, but a 2.6 kb band indicated Zm6-*ndh*.

Primers for PCRs were supplied by Operon and Invitrogen. PCRs were carried out in an Eppendorf Mastercycler, using Fermentas Taq DNA polymerase. Other DNA manipulations were carried out as described previously [8, 10], using Qiagen kits, and following manufacturer’s instructions. All DNA constructs were confirmed by DNA sequencing, carried out by Beckman Coulter genomics.

### Cultivation and incubation of non-growing cells

Batch cultivations were carried out in a thermostat at 30°C, either in 50 ml Falcon tubes or in 200 ml flasks. Growth medium contained 20 g L^-1^ glucose, 5 g L^-1^ yeast extract and mineral salts, as described in [8]. For preparation of non-growing *Z*. *mobilis* suspension, cells were harvested at late exponential growth phase, sedimented by centrifugation at 5000 r.p.m. for 15 min, washed, and resuspended in 100 mM potassium phosphate buffer (pH 6.9), containing 2 mM magnesium sulfate, to biomass concentration of 4.0 g dry wt L^-1^. Experiments with non-growing *Z*. *mobilis* cell suspensions were carried out in a Biostat Q-plus, Sartorius tabletop fermenter, 200 ml working volume, at 30°C. In order to ensure hyperventilation (for efficient withdrawal of volatile products), fermenter was gassed with either N_2_ or atmospheric air, maintaining stirring rate at 500 r.p.m. The gas flow was kept at 0.5 L min^−1^, so that the amount of gas passing through the system per one minute 2.5 times exceeded the working volume of the fermenter. Samples were collected every 5 or 10 minutes, both during the anaerobic and aerobic phase of the experiments.

### Protein extraction and SDS-PAGE

Protein extraction and SDS-PAGE were performed following a slightly modified procedure of Sootsuwan *et al*., [33]. Cells were harvested by centrifugation, washed, and resuspended in 10 mM Tris HCl (pH 7.0) buffer. Proteins were extracted by sonication in an UP-200s (Dr. Hielscher, Germany) ultrasonic processor for 5 minutes with pulses of 0.5-s duration, separated by 0.5-s intervals. 40 μg of the protein sample was heated at 100°C for 5 minutes and separated by SDS electrophoresis on 12% acrylamide gel. Gel was visualized using Coomassie Brilliant Blue R250.

### Monitoring of enzyme activities

Alcohol dehydrogenase activity (the total activity of both isoenzymes, ADH I and ADH II) was monitored in permeabilized cells, as described in [34]. Other enzyme activities were measured in cell-free extract. For preparation of cell-free extract, cells were sedimented by centrifugation at 5,000 rpm for 15 min, washed, and resuspended in 100 mM potassium phosphate buffer, containing 2 mM magnesium sulfate, pH 6.9, to an OD_550_ of about 20, corresponding to a biomass concentration of approx. 4 mg (dry wt) ml^-1^. Cells were broken by a 2-min ultrasonic treatment with pulses of 0.5-s duration, separated by 0.5-s intervals, using a Dr. Hielscher ultrasonic processor. Subsequent removal of unbroken cells was performed as before [10]. The activity of respiratory NAD(P)H dehydrogenase (Ndh) was measured as the decrease of NADH absorbance at 340 nm using Nanodrop 2000C, Thermo Scientific spectrophotometer. Activities of the rest of enzymes were measured in the supernatant of the cell-free extract, after sedimentation of membrane fraction by ultracentrifugation [10]. Pyruvate decarboxylase was monitored by following NADH oxidation in the presence of pyruvate, NADH, and alcohol dehydrogenase, as described in [35]. Glucose-6-phosphate dehydrogenase [35] and Glyceraldehyde-3-phosphate dehydrogenase [36] were monitored by recording NAD^+^ reduction rate at 340 nm in the presence of the respective substrates.

### Analytical methods

Glucose and ethanol concentrations were measured by HPLC (Agilent 1100 series), using a Biorad Aminex HPX-87H column. Alternatively, glucose consumption was monitored following CO_2_ concentration in the outlet gas of the fermenter, using an Infors HT Gas analyser (Switzerland). Intracellular concentration of phosphoenolpyruvate (PEP) was determined with high resolution mass spectroscopy (HRMS) in cell lysates quenched by perchloric acid. Spectra were taken using Agilent 6230 TOF LC/MS system (Agilent Technologies, Germany) with positive electrospray ionization. Samples for ATP determination were quenched in ice-cold 10% trichloroacetic acid and assayed by the standard luciferin-luciferase method using a TECAN Infinite 200Pro microplate reader. NAD(P)H intracellular concentration in non-growing *Z*. *mobilis* cell suspensions was continuously monitored on-line as fluorescence (excitation at 340 nm, emission at 460 nm) using a Varian fluorimeter [37]. Cell suspension was constantly pumped through a closed circuit ensuring time lag of 4 seconds between the working volume of fermenter and the fluorimeter cuvette. In parallel, NAD(P)H concentration in quenched samples was determined by a NAD(P)H bioluminescence kit from Roche diagnostics, as in [34] using a TECAN Infinite 200Pro microplate reader. Oxygen uptake rate was calculated from the data obtained by on-line measurements of oxygen in the inlet and the outlet gas streams of the fermenter using gas analyser (Infors HT). Cell concentration was monitored as the optical density OD_550_, and the dry cell mass of suspensions was calculated by reference to a calibration curve.

### Metabolic modeling

The kinetic model of *Z*. *mobilis* E-D pathway that was used in this study has been described and validated previously [32]. In order to simulate the interplay between E-D pathway and NADH withdrawal by respiratory chain, we added reactions describing NAD(P)H oxidation (NDH) and ATP synthesis via oxidative phosphorylation (OXP). The rate of NDH was calculated from oxygen analysis data in the fermentor outlet gas, and used in the model as a simple NAD(P)H sink reaction, taking the NAD(P)H to O_2_ ratio for *Z*. *mobilis* respiration 2:1 (see [24]). To describe the rate of oxidative phosphorylation, the rate of NAD(P)H oxidation was multiplied by P/O ratio, assuming its value either 0 (absence of oxidative phosphorylation) or 1 (oxidative phosphorylation of moderate efficiency). To avoid the ‘model explosion’ of the intracellular acetaldehyde pool as a consequence of NADH withdrawal by the respiratory chain, we introduced an acetaldehyde export reaction (AEX). The kinetic expression was derived by approximating to previously reported experimental measurements [38]. The rate equations used in all newly introduced reactions, as well as the source of the parameter values are given in S1 Table. As previously, kinetic modeling and metabolic control analysis (MCA) was carried out using COPASI software [32]. The control coefficients of enzymes over the glycolytic flux were expressed as percentages, summing up to 100% (see S2 Table).

## Results

### Transition from anaerobic to aerobic glucose consumption under steady-state

The published kinetic model of the *Z*. *mobilis* E-D pathway ([32]; https://www.ebi.ac.uk/biomodels-main/MODEL1409050000) simulates steady-state fluxes and intermediate concentrations in non-growing cells in a reasonably good agreement with the reported experimental studies by ^31^P-NMR in dense anaerobic glucose-consuming suspensions, yet it does not involve respiratory reactions. The model includes reactions for all E-D pathway enzymes, pyruvate decarboxylase, both alcohol dehydrogenase isoenzymes, adenylate kinase, as well as a lumped reaction for ATP consumption in biosynthesis, maintenance, and dissipation, assuming simple hyperbolic kinetics. For purpose of this study, we extended the model by introducing: (i) a respiratory NADH oxidation reaction with a constant rate, corresponding to the measured steady-state oxygen uptake for non-growing, glucose-consuming cells, (ii) a reaction for oxidative phosphorylation, assuming P/O stoichiometry values between 0 and 1, and (iii) a reaction of acetaldehyde export from cells (this product was not included in the previously published model, because it accumulates only under aerobic conditions; see Fig 1) (S1 Table).

The extended version of the model was used for simulation experiments. Anaerobic (non-respiring) steady-state with model parameters as in [32] was perturbed *in silico* by onset of the NADH oxidation reaction. The simulation results were compared to the respective steady-states obtained *in vivo* with glucose-consuming, non-growing cell suspension. The results are presented in Tables 1 and 2.

**Table 1:** Steady-state flux parameters of Zm6 non-growing cell suspension, and the results of the E-D model simulation for anaerobic and aerobic conditions.

	q _glucose_		q_ethanol_		q_O2_
	Anaerobic	Aerobic	Anaerobic	Aerobic	Aerobic
Glucose-consuming non-growing cells	2.6 ± 0.3	3.5 ± 0.4	1.1 ± 0.2	1.2 ± 0.1	4.3 ± 1.6
Model simulation, P/O = 0	4.9	5.4	2.5	2.6	4.3
Model simulation, P/O = 1	4.9	4.7	2.5	2.2	4.3

Errors represent ± SD (n≥5). Specific rates of glucose consumption and ethanol production are presented as g · (g DCW · h)^-1^, that of oxygen consumption is given as mmol · (g DCW · h)^-1^; DCW, dry cell weight.

**Table 2:** Intracellular concentration (μM) of key metabolic intermediates under anaerobic and aerobic steady state.

	Experimental		Model		
Intermediate	Anaerobic	Aerobic	Anaerobic*	Aerobic**	Aerobic***
PEP	450 ± 123	961 ± 290	66	241	268
NAD(P)H	1334 ± 61	186 ± 22	2983	3	2
ATP	1601 ± 187	3042 ± 380	1671	2929	3233

Model simulation data:

* results from [32],

** simulation assuming P/O = 0,

*** simulation assuming P/O = 1. Errors represent ± SD (n = 3).

In non-growing cell suspension glucose uptake proceeded with a constant rate, and steady levels of intracellular ATP, NAD(P)H and PEP were established. When nitrogen gas was replaced by air, cells rapidly acquired a new steady-state, in which the intracellular NAD(P)H level had decreased, while the specific rate of glucose consumption (q _glucose_) had increased significantly (P<0.01), by about 35% (Table 1), and the intracellular ATP concentration had almost doubled (Table 2). Also, the concentration of PEP, an allosteric inhibitor of glucose-6-phosphate dehydrogenase [39] had doubled, hence ruling out the possibility that the aerobic rise of q _glucose_ might result from a release of the PEP inhibitory effect. Model simulation, assuming P/O = 0, reproduced the directionality of all these effects, although the predicted relative increase of q _glucose_ was smaller than experimentally observed, the fall of NAD(P)H concentration was more pronounced, and PEP concentration increased by a factor of four, yet the simulated concentrations were below the experimental values. Notably, the feedback inhibition mechanism by PEP had been included in the original E-D model [32], so that the model predicted an increase of q _glucose_, taking into account the inhibitory effect of the elevated PEP concentration.

**Fig 2:**
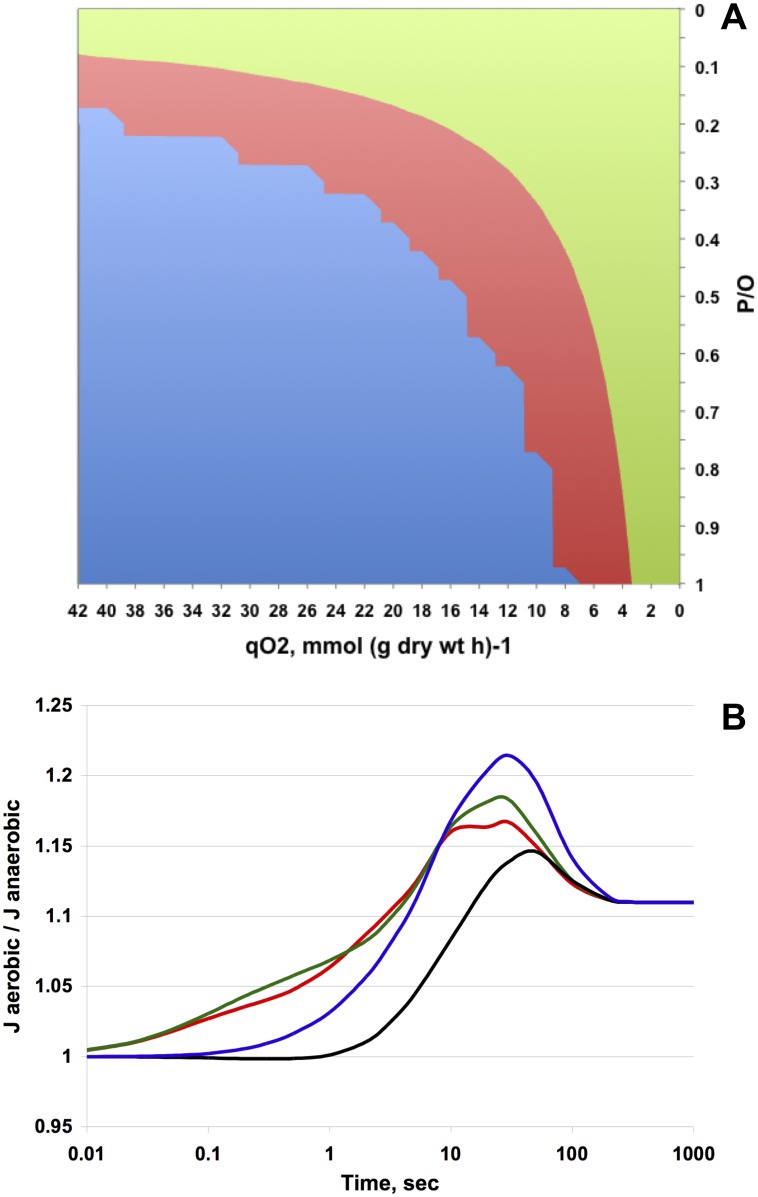
Model simulation of the E-D pathway fluxes. (A) Dependence of the ratio between the aerobic and anaerobic steady-state glucose consumption rates (q _gluc aer_ / q _gluc anaer_) on the P/O stoichiometry and oxygen consumption rate. Green area, (q _gluc aer_ / q _gluc anaer_) within the range 1.00–1.11; red area, (q _gluc aer_ / q _gluc anaer_) within the range 0.85–1.00; blue area, model has no steady-state solutions. (B) The time-course of the relative rates (J_aerobic_/J_anaerobic_) of the ATP/ADP or NADH/NAD^+^-involving E-D pathway reactions during transition from anaerobic to aerobic steady state. The NADH-oxidising reaction (‘sink’), corresponding to the experimentally observed steady state oxygen consumption rate, is turned on at zero time; P/O = 0. Red line–GAPDH and PGK, green line—G6PD, blue line—GK, black line—PYK.

The model results for steady state intracellular ATP concentration (Table 2) matched the experimental observations fairly well for both P/O values. As expected, simulations with P/O = 1 gave higher aerobic ATP due to contribution of oxidative phosphorylation. However, that occurred in combination with a decrease of q _glucose_ (Table 1), contradicting our *in vivo* evidence. Simultaneous increase of ATP concentration and glucose uptake rate could be achieved *in silico* only by assuming respiration with P/O close to zero. Fig 2A represents screening of the model simulation results for the effect of P/O and the oxygen consumption rate on the steady state ratio between the aerobic and anaerobic rate of glucose uptake. Simulation showed that the aerobic increase of the E-D pathway flux would take place only at low P/O, and that this P/O threshold value would decrease along with increase of the specific oxygen consumption rate. At oxygen uptake rate of 4.3 mmol · (g DCW · h)^-1^ (Table 1) aerobic stimulation of the E-D pathway flux would happen only with P/O below 0.7. Thus, according to the model results, respiration in glycolyzing *Z*. *mobilis* cells was either devoid of phosphorylation, or proceeded with a low P/O stoichiometry, and interacted with the E-D pathway primarily by lowering the intracellular NADH/NAD^+^ ratio. Accordingly, the metabolic control analysis (MCA) with the model (S2 Table) showed that the lumped ATP-consuming reaction contributed the major part of control over the E-D flux, and its control coefficient further increased after transition to respiring conditions.

### Effect of Ndh activity on the initial rate of glucose uptake in non-growing cell suspension

**Fig 3:**
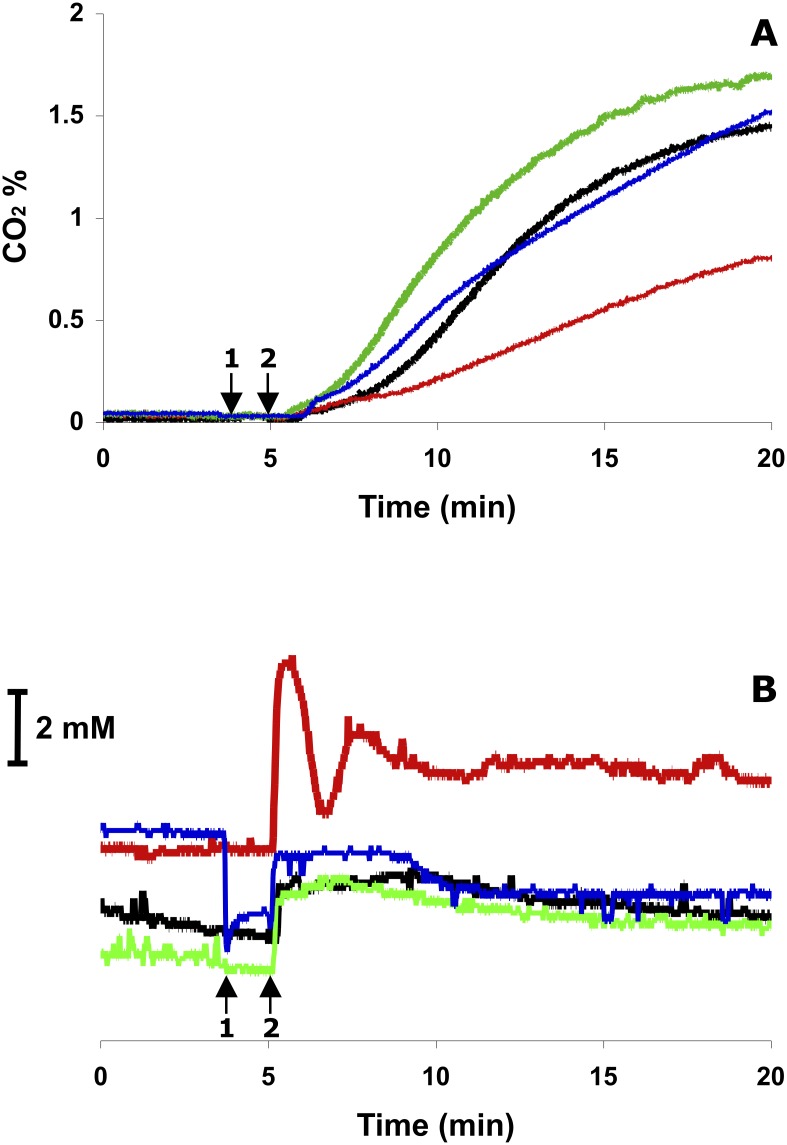
Glucose uptake in non-growing cell suspensions of Zm6 (black line), Zm6-*ndh* (red line), Zm6-*ndh*+ (green line), and Zm6-*ndh* with acetaldehyde (blue line). Arrows denote: (1) addition of acetaldehyde to Zm6-*ndh*, (2) addition of glucose to all variants. (A) Initial rate of glucose utilization, monitored by recording of CO_2_ production after glucose addition. (B) Time course of the intracellular NAD(P)H after glucose addition. The scale bar refers to the shift of fluorescence intensity, which would correspond to the change of intracellular NAD(P)H concentration by 2mM, taking that at cell concentration of 4 mg DCW/ml the intracellular volume constitutes 0.9% of the total volume of suspension. A representative experiment is shown.

Acceleration of the E-D pathway flux by respiratory chain activity added a novel facet to the physiological role of respiration in *Z*. *mobilis*, implying that the aerobic glucose uptake should differ also between the wild type and respiratory mutants. Therefore, respiratory mutants were the next focus of our study. We compared initial rates of glucose uptake after glucose addition to non-growing cells of strains Zm6, Zm6-*ndh* and Zm6-*ndh*+. Since the Krebs cycle in *Z*. *mobilis* is truncated and does not function as a catabolic pathway, the pyruvate decarboxylase reaction is the only source of CO_2_ in glucose-consuming cells. That allows to monitor the E-D pathway flux continuously by recording CO_2_ evolution. In these experiments with aerated cell suspensions glucose consumption was followed on-line by recording CO_2_ concentration in the fermenter outlet gas. Simultaneously, the relative change of NAD(P)H concentration was monitored by fluorimetry. Results are shown in Fig 3. We observed a large difference in CO_2_ evolution between the wild type strain Zm6 and the Ndh knock-out mutant. Zm6-*ndh* produced CO_2_ at a rate that was less than half of the average rate, typical for Zm6 during the period of the first minutes after glucose addition (Fig 3A). At the same time, glucose consumption in the complemented strain Zm6-*ndh*+ was comparable to that in Zm6. The glucose consumption rate strictly correlated with the presence of functional Ndh and the respiratory NADH oxidase activity. Zm6-*ndh* showed a near-zero activity of NADH oxidase, while the complemented mutant strain, carrying *ndh* under control of its own promoter in the plasmid pBBRndh, expressed a higher activity than in the wild type (see S3 Table). Notably, the closely similar SDS-PAGE pattern of the soluble proteins for all three strains within the range of the apparent molecular weights from 59 kDa (pyruvate decarboxylase) to 22 kDa (2-keto-3- deoxy-6-phosphogluconate aldolase), which includes all the E-D pathway and ethanologenic enzymes [40], indicated that neither inactivation nor overexpression of *ndh* had any substantial side effects on the expression of the glucose catabolic pathway (see S1 Fig). This was also confirmed by the fact that activities of glucose-6-phosphate dehydrogenase, pyruvate decarboxylase, alcohol dehydrogenase and glyceraldehyde-3-phosphate dehydrogenase did not differ significantly between the parent strain and any of the mutants (S3 Table).

In Zm6, glucose addition caused an almost immediate rise of NAD(P)H fluorescence, which gradually decreased after 5 minutes, but remained well above the initial level (Fig 3B). The estimated increase of intracellular NAD(P)H concentration in response to glucose addition reached values between 1 and 2 mM. That was within the same order of magnitude as the concentration decrease recorded by luminescence assay during transition from anaerobic to aerobic conditions (Table 2). However, in Zm6-*ndh* cells, fluorescence was substantially higher than in Zm6 cells, both before and after glucose addition. When glucose was added, fluorescence spiked, and the new steady state was reached via damped oscillations. Apparently, the respiratory chain in *Z*. *mobilis* wild type contributed to stabilizing of the NAD(P)H concentration and smoothening of perturbations. Complementation of the mutant restored the dynamics of NAD(P)H fluorescence typical for Zm6, as observed also for CO_2_ evolution.

To support our conclusion that respiration stimulated the E-D pathway flux by lowering the intracellular NADH/NAD^+^ ratio, we used an alternative means to oxidize the intracellular NADH in Zm6-*ndh*. Shortly before glucose addition, acetaldehyde was added to Zm6-*ndh* cell suspension at final concentration of 10 mM. Acetaldehyde freely penetrates *Z*. *mobilis* cells and is used as the substrate of alcohol dehydrogenase reaction when NADH is also available (see Fig 1). Although acetaldehyde inhibits growth and biosynthesis, the catabolic pathway is much less sensitive to it [21, 22]. Therefore, we used the alcohol dehydrogenase reaction with externally added acetaldehyde for NADH oxidation as a substitute for the respiratory chain in Zm6-*ndh*. As seen in Fig 3B, addition of acetaldehyde immediately decreased NAD(P)H fluorescence in Zm6-*ndh*. The fluorescent response to glucose addition shortly afterwards resembled very much the time-course of fluorescence in strains Zm6 and Zm6-*ndh*+. With acetaldehyde, CO_2_ evolution in Zm6-*ndh* started earlier, proceeded faster, and its rate was comparable to that in Zm6 (Fig 3A). At the same time, addition of acetaldehyde to Zm6 had negligible effects.

### The competitive advantage of respiring *Zymomonas mobilis* over the non-respiring Ndh-negative strain

**Fig 4:**
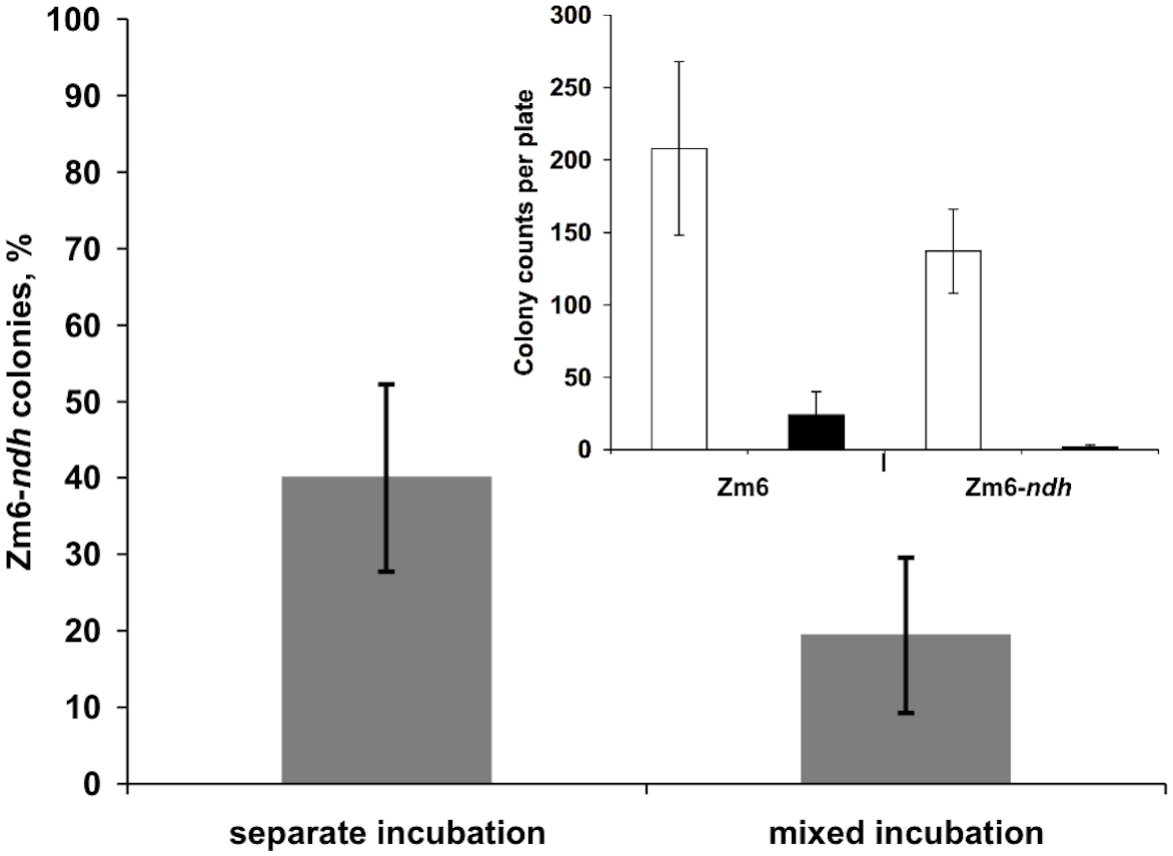
Survival rate of Zm6-*ndh* in mixed platings with Zm6, after 8 hours of mixed or separate incubation of strains in phosphate buffer with periodical glucose feed. Inset—colony counts of separately incubated Zm6 and Zm6-*ndh*, with periodical glucose addition (empty bars), or without glucose addition (filled bars). Data represent mean values of 5 (mixed platings with separate incubation), 8 (mixed incubation), or 6 (inset; separate platings) independent experiments; error bars represent SD. The P value of the difference between the results of separate and mixed incubations was found to be 0.007. The P value of the difference between colony counts of separately incubated Zm6 and Zm6-*ndh* with periodical glucose addition was 0.027.

Based on the above results, we hypothesized that an active respiratory chain may be advantageous for reaching a high glucose uptake rate in non-growing cells, in order to survive when glucose is available in small amounts and for restricted periods of time. In the paper of Dawes and Large [41] it was shown that in starving *Z*. *mobilis* viability decreased exponentially as soon as starvation began, accompanied by a corresponding decrease in intracellular ATP, which was most marked during the initial 6–8 hours. When starved cells were supplied with glucose, the intracellular ATP pool was restored [41]. In line with these observations, here we demonstrated that periodic glucose addition improved the viability of non-growing *Z*. *mobilis*. We compared colony counts of strains Zm6 and Zm6-*ndh*, plated after 8 h of aerobic incubation of cells in phosphate buffer, both with and without regular supplements of glucose (Fig 4, inset). Bacteria were grown overnight, the cells were washed and resuspended in potassium phosphate buffer to the concentration of 2 g dry wt L^-1^. Then, 3 ml aliquots of the suspension were transferred into 50 ml Falcon tubes and incubated on a shaker at 90 r.p.m., 30°C, for 8 hours. After the first hour of incubation, glucose solution was added to give 1 g L^-1^ glucose final concentration in the suspension, and such additions were repeated every hour. Glucose was taken up by the cells within several minutes, so that it did not accumulate in the medium during the experiment. Control samples did not receive glucose. At the end of the 8 hour incubation, all samples were diluted in sterile distilled water by a factor of 4 · 10^4^, and 10 μl were plated for colony counts. As seen in Fig 4 (inset), glucose supplements had a strong positive effect on the viability of both strains. For Zm6, glucose supplements improved colony counts by about an order of magnitude. For the survival of Zm6-*ndh*, the requirement for glucose was even stronger than for Zm6: very few Zm6-*ndh* colonies appeared on control plates.

At the end of the 8-hour incubation, when equal volumes of the glucose-fed cell suspensions of both strains were mixed together and plated immediately, colony PCR with *ndh* primers indicated that approximately 40% of the emerging colonies were Zm6-*ndh* (Fig 4). This percentage was in a good agreement with the colony counts for separately plated glucose-fed strains (compare the empty bars in the inset): the glucose-fed Zm6 yielded an average of about 210 colonies per plate, while Zm6-*ndh* yielded close to 140 colonies. Notably however, when both strains were mixed in the same proportion already at the beginning of the 8-hour incubation, whereby that the hourly glucose feed was supplied into a mixed cell population, later on plates less than 20% of colonies were identified as Zm6-*ndh*. These data indicate an adaptive advantage of Zm6 over Zm6-*ndh* under conditions where rapid uptake of limited glucose amount is important for survival. The respiring strain Zm6 manages to restore its energy reserve more completely, since its initial rate of glucose catabolism is higher. So, the cells of Zm6 could benefit from consuming most of the added glucose, and thus be better prepared to enter the following period of starvation.

## Discussion

In the present work we demonstrated that in non-growing cells of *Zymomonas mobilis* respiration accelerates glucose consumption. In the wild type strain Zm6, the specific rate of glucose uptake increased after transition from anaerobic to aerobic conditions. Also the rate of glucose consumption was shown to be higher in aerated cell suspension of the wild type than in the aerated non-respiring Ndh-deficient strain, particularly during the first few minutes after glucose administration. Since glucose enters *Z*. *mobilis* cells via an energy-independent facilitator (Glf) [42], we hypothesized that the target for the respiratory activation lies downstream the transport step, within the E-D pathway itself. Monitoring the intracellular PEP concentration ruled out the possibility that the E-D flux might increase due to an aeration-dependent fall of the PEP concentration, and a concomitant release of the glucose-6-phosphate dehydrogenase inhibition. In principle, respiration could act upon the E-D pathway in several ways, affecting both the cellular energy state and the reducing equivalent balance, hence the mechanism beyond these simple effects seemed far from obvious. Therefore, we applied quantitative modeling tools for analysis.

Using the E-D pathway kinetic model, it was possible to reproduce the directionality and the order of magnitude of the observed effects of respiration *in silico*, although some of them without quantitative precision. The simulations clearly indicated that the oxidation of extra NAD(P)H *per se*, but not oxidative phosphorylation, was the driving force behind the acceleration of the E-D pathway flux and rise of intracellular ATP concentration. Although being in agreement with the previously observed low energetic efficiency of respiration in *Z*. *mobilis*, this conclusion might seem somewhat counterintuitive. Based on plain ‘common sense’, instead of modeling, a plausible alternative can be raised: oxidative phosphorylation (assuming that it occurs with low P/O) slightly increases ATP production immediately after the start of aeration, and hence adds momentum to the E-D pathway flux by supplying more ATP to the glucokinase reaction. Model simulations, however, showed that (i) in glucose-consuming cells oxidative phosphorylation, even with a modest P/O, did not stimulate, but rather slowed down the E-D flux, and (ii) the elevated ATP level was not the cause, but the consequence of the increased E-D flux. If the oxidative phosphorylation would be the driving force behind the acceleration of the E-D pathway flux, then one would expect a positive correlation of the glucose consumption rate with the P/O ratio. According to the model, that was not the case. Vice versa, the highest aerobic acceleration of the E-D pathway flux took place at P/O = 0, remaining still below the experimental value (Table 1). Accordingly, the model’s MCA data showed a high positive control over the E-D pathway flux by the ATP dissipating reactions and negative control by oxidative phosphorylation (S2 Table). In addition, it was demonstrated experimentally that oxidation of NAD(P)H by respiration was not the only way to increase the E-D flux. Establishing an alternative sink of NADH via the alcohol dehydrogenase reaction elevated the glucose consumption in the non-respiring mutant strain Zm6-*ndh* up to the wild type level.

We speculated that the glyceraldehyde-3-phosphate dehydrogenase (GAPDH) was a potential site in the E-D pathway, which initially could respond to decrease of the NADH/NAD^+^ ratio. This reaction belongs to the ‘thermodynamic bottleneck’ of the E-D pathway [43], and thus can be substantially accelerated by the removal of one of its products, NADH. Simulation of the transition from the anaerobic to aerobic steady state showed (Fig 2B) that both GAPDH, and the other NAD(P)H-generating reaction of the E-D pathway, glucose-6-phosphate dehydrogenase (G6PDH), almost instantly responded to the onset of the respiratory NAD(P)H ‘sink’ reaction. Importantly, the transition kinetics of GAPDH coincided with that of the phosphoglycerate kinase (PGK). The PGK reaction thus appeared to be the immediate source of extra ATP produced upon the onset of aeration.

Notably, response of a growing culture to aeration strikingly differs from that of non-growing cells, as described in the present study. For growing *Z*. *mobilis* cultures, several papers have reported a variety of effects of aeration on glucose consumption, including a slight stimulation, inhibition, or lack of any change at all [23, 24, 44, 45]. Although the underlying mechanisms for this major difference are not clear, it is obvious that *Z*. *mobilis* does not use its respiratory chain to supply energy for aerobic growth in the same way as the majority of aerobic and facultatively anaerobic microorganisms do. Still, the very presence of an active respiratory chain naturally implies that respiration is advantageous at some time during the life cycle. Our results suggest that these times might be when starving cells of *Z*. *mobilis* obtain access to a few drops of some sugary plant sap, and the speed of glucose uptake then determines their chance of further survival. This novel type of respiratory function is not well manifested under routine laboratory cultivation and maintenance on rich media, yet it might be relevant for overcoming hardships in nature.

## Supporting Information

S1 Fig SDS-PAGE analysis of proteins isolated from *Z*. *mobilis* strains: Zm6, Zm6-*ndh*, Zm6-*ndh+*. (TIFF)

S1 Table Rate equations and kinetic parameters of the additional model reactions used in this study. V_NDH_, V_OXP_, V_AEX_—the specific activities of the reactions describing NADH oxidation, oxidative phosphorylation and acetaldehyde export. V_f_—maximum rate of the forward reaction [μmol (L s)^-1^]. P/O—molar ratio of oxidative phosphorylation (synthesized molecules of ATP / reduced oxygen atoms). ACETcy, ACETex,–acetaldehyde concentration [μmol L^-1^] in the cytoplasm and extracellular space, accordingly.
(TIFF)

S2 Table Scaled flux control coefficients CJ_i_ of the glycolytic flux in the E-D pathway under anaerobic and aerobic conditions expressed as percentage. Control coefficients above 1% are shown in bold type.
(TIFF)

S3 Table Activities of the respiratory NAD(P)H dehydrogenase and several enzymes of the ethanologenic pathway [U (mg protein)^-1^] in cell-free extracts or in permeabilized cells (alcohol dehydrogenase) of the strains Zm6, Zm6-*ndh* and Zm6-*ndh+*. (TIFF)
