# Supplementary figures and images for: The Low Energy-Coupling Respiration in Zymomonas mobilis Accelerates Flux in the Entner-Doudoroff Pathway
Source: PLoS One. 2016 Apr 21;11(4):e0153866. doi: 10.1371/journal.pone.0153866 (PMC4839697; doi:10.1371/journal.pone.0153866)

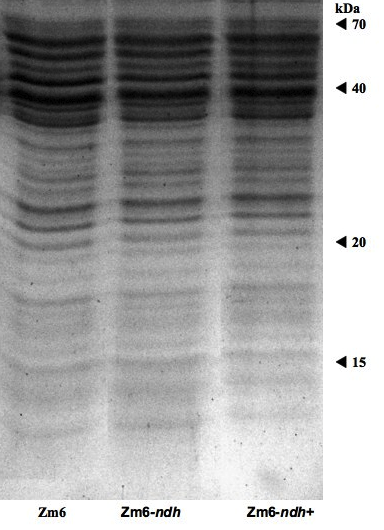

Supplement: S1 Fig — (TIFF) [file pone.0153866.s001.tiff]

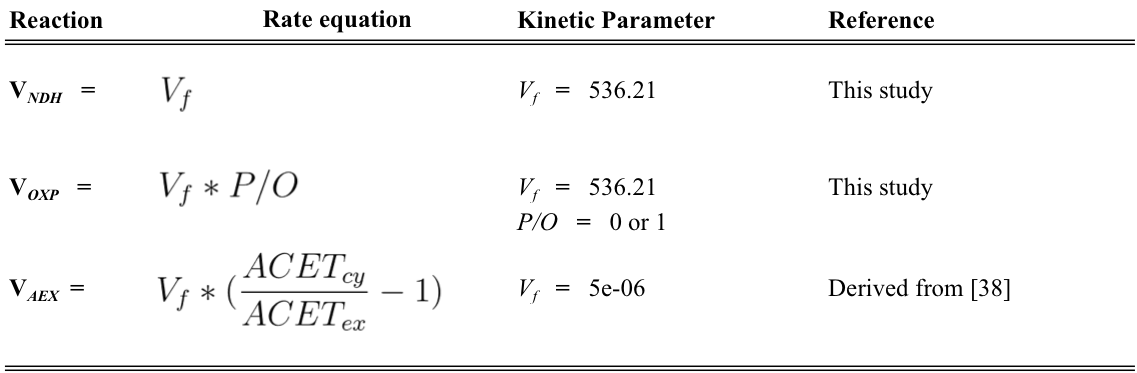

Supplement: S1 Table — VNDH, VOXP, VAEX—the specific activities of the reactions describing NADH oxidation, oxidative phosphorylation and acetaldehyde export. Vf—maximum rate of the forward reaction [μmol (L s)-1]. P/O—molar ratio of oxidative phosphorylation (synthesized molecules of ATP / reduced oxygen atoms). ACETcy, ACETex,–acetaldehyde concentration [μmol L-1] in the cytoplasm and extracellular space, accordingly. (TIFF) [file pone.0153866.s002.tiff]

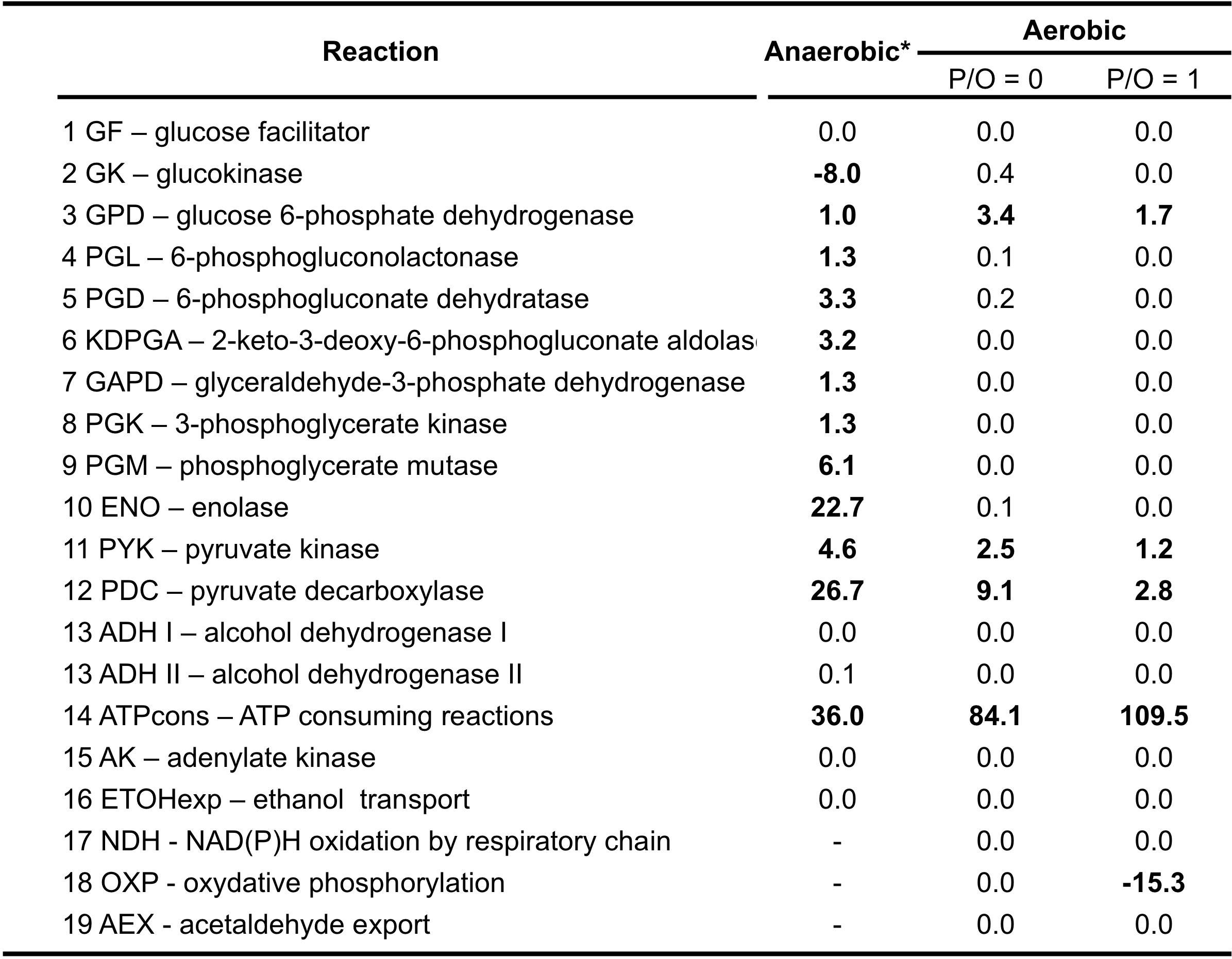

Supplement: S2 Table — Control coefficients above 1% are shown in bold type. (TIFF) [file pone.0153866.s003.tiff]

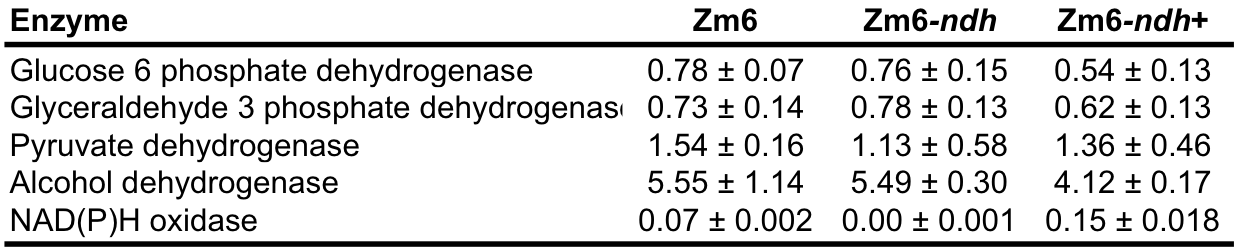

Supplement: S3 Table — (TIFF) [file pone.0153866.s004.tiff]
